# Prevalence and factors associated with atrial fibrillation in older patients with obstructive sleep apnea

**DOI:** 10.1186/s12877-022-02791-4

**Published:** 2022-03-14

**Authors:** Huanhuan Wang, JianHua Li, Yinghui Gao, Kaibing Chen, Yan Gao, JingJing Guo, Min Shi, Xiao Zou, Weihao Xu, LiBo Zhao, Xiaofeng Su, Yabin Wang, Juan Liu, Hu Xu, Xiaoxuan Kong, Junling Lin, Xiaoshun Qian, Jiming Han, Lin Liu

**Affiliations:** 1 Department of Pulmonary and Critical Care Medicine of the Second Medical Center, Chinese PLA General Hospital, Beijing, China; 2 Medical College, Yan’an University, Yan’an, Shaanxi Province China; 3 Cardiology Department of the Second Medical Center and National Clinical Research Center for Geriatric Diseases, Chinese PLA General Hospital, Beijing, China; 4 PKU-UPenn Sleep Center, Peking University International Hospital, Beijing, China; 5 Sleep Center, the Affiliated Hospital of Gansu University of Chinese Medicine, Lanzhou City, Gansu Province China; 6 Department of General Practice, 960Th Hospital of PLA, Jinan, Shandong Province China; 7 Sleep Medicine Center, Department of Respiratory and Critical Care Medicine, Peking University People’s Hospital, Beijing, China; 8 Department of Respiratory and Critical Care Medicine, Beijing Chaoyang Hospital Affiliated To Capital Medical University, Beijing, China

**Keywords:** Obstructive sleep apnea, Atrial fibrillation, Older people, Prevalence, China

## Abstract

**Purpose:**

This study sought to identify the prevalence and factors associated with atrial fibrillation (AF) in older patients with obstructive sleep apnea (OSA) in China.

**Methods:**

This was an explorative cross-sectional study. Between January 2015 and October 2017, we continuously recruited 1285 older patients with OSA who underwent overnight polysomnography from sleep centers of multiple hospitals. They were assessed using 12-lead ECG or 24-h dynamic ECG, and their baseline demographics, clinical characteristics, sleep parameters, and medical history were determined. Multivariate binary logistic regression analysis was used to investigate the factors related to AF in these older patients with OSA.

**Results:**

The clinician classified 122 (9.5%) patients as having AF. The prevalence of AF significantly increased with age (*P* < 0.05) but did not significantly differ between the mild, moderate, and severe OSA groups. Additionally, the prevalence of paroxysmal AF was 7.2% among the overall study population, and it increased with OSA severity or advanced age (*P* < 0.05). Persistent AF was noted in 2.3% participants, and the prevalence also increased with age. The logistic regression analysis showed that age (OR = 1.054, 95%CI: 1.027–1.018, *P* < 0.001), history of drinking (OR = 1.752, 95%CI: 1.070–2.867, *P* < 0.05), chronic heart disease (OR = 1.778, 95%CI: 1.156–2.736, *P* < 0.01), diabetes mellitus (OR = 1.792, 95%CI: 1.183–2.713, *P* < 0.01), and reduced diastolic function (OR = 2.373, 95%CI = 1.298–4.337, *P* < 0.01) were relevant to AF among participants with OSA.

**Conclusion:**

The prevalence of AF is significantly common in older patients with OSA. Age, history of drinking, chronic heart disease, diabetes mellitus, and reduced diastolic function are independently related to AF in these patients.

## Introduction

Obstructive sleep apnea (OSA), the most common type of sleep apnea, is an independent risk factor for cardiovascular diseases and all-cause mortality [1–3]. Moreover, patients with OSA were frequently diagnosed with hypertension [4], diabetes mellitus (DM) [5], and stroke [6]. Several clinical studies have revealed that OSA is highly prevalent (60–90%) in patients with atrial fibrillation (AF) [7–9]. OSA promotes the occurrence and recurrence of AF, which is the most common clinically observed form of arrhythmia, due to the repetitive cycles of intermittent hypoxia causing an imbalance of cardiac autonomic modulation [9]. Moreover, older patients with OSA are more prone to developing AF [10, 11].

The prevalence of OSA among patients with AF, including those who underwent catheter ablation has been reported. However, the prevalence and factors associated with AF in OSA patients are not clearly studied. Therefore, we performed this large-scale, explorative cross-sectional study to assess the prevalence and risk factors of AF among older patients with OSA.

## Methods

### Study Population

**Fig. 1:**
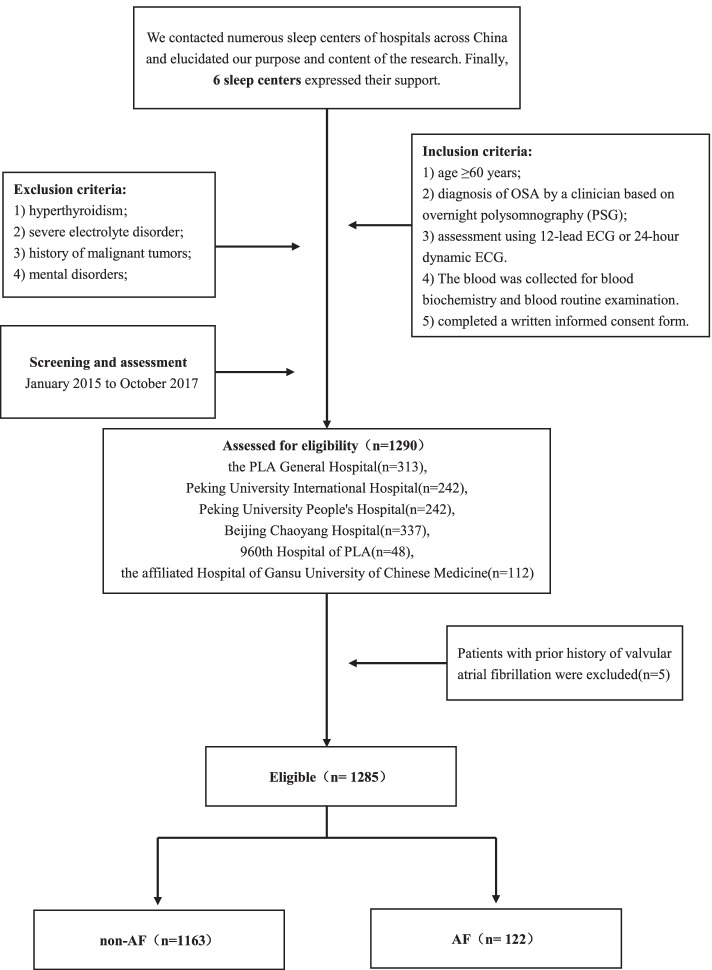
Study flowchart. OSA: obstructive sleep apnea; PSG: polysomnography; ECG: electrocardiography; AF: atrial fibrillation

This is an explorative observational multi-center study, a total of 1290 patients were continuously recruited from the PLA General Hospital(*n* = 313), Peking University International Hospital(*n* = 242), Peking University People's Hospital(*n* = 242), Beijing Chaoyang Hospital(*n* = 337), 960th Hospital of PLA(*n* = 48), and the affiliated Hospital of Gansu University of Chinese Medicine(*n* = 112) between January 2015 and October 2017. Our inclusion criteria were as follows:1) age ≥ 60 years; 2) diagnosis of OSA by a clinician based on overnight polysomnography; 3) assessment using 12-lead ECG or 24-h dynamic ECG; 4) The blood was collected for blood biochemistry and blood routine examination. The exclusion criteria were as follows: hyperthyroidism, severe electrolyte disorder, history of malignant tumors; and mental disorders. The study flowchart is presented in Fig. 1. Immediately after, the subjects with prior history of valvular atrial fibrillation (*n* = 5) were also excluded according to our study purpose. Ultimately, 1285 older patients with OSA were included in the analysis, and any ethnic heterogeneity didn’t exist among participants due to that they were both *Han* Chinese. This study was carried out in accordance with the Declaration of Helsinki. The ethics committee of PLA General Hospital approved the study (S2019-352–01). Written informed consent was available from all participants.

### Baseline Evaluation

Demographic data and clinical characteristics of all patients were collected by the researchers, including sex, age, nationality, height, weight, body mass index [BMI was defined as weight in kilograms divided by height in meters squared and was expressed in units of kg/m2. According to the WHO definition: obese, BMI ≧ 30; overweight, 25 ≤ BMI < 30; normal weight, 18.5 ≤ BMI < 25; underweight, BMI < 18.5], blood pressure, history of smoking (was ascertained on the basis of self-reported history of cigarette smoking, according to whether smoking consecutive or accumulated more than six months or not) [12], and history of drinking (was defined as drinking at least once a week over a year, and currently having drank or quit drinking for less than three years). As recommended by current guidelines, the E/A ratio is the ratio of the early (E) to late (A) ventricular filling velocities, and reduced diastolic function was defined as: E/A ≤ 0.7 or deceleration time (DT) > 260 ms; or E/A between 0.7–1.5 and peak early diastolic velocity (e′) < 7 cm/s; or E/A > 1.5 and e′ < 7 cm/s or DT < 140 ms [13]. as well as data on sleep parameters [including the apnea–hypopnea index (AHI), the oxygen desaturation index (ODI), total sleep time (TST) the time under saturation of 90%, the mean pulse oxygen saturation(MSpO2), the lowest pulse oxygen saturation(LSpO2)], blood index (blood of participants were collected under the conditions of at least 6 h of fasting and 4 h of drinking) included total bilirubin, direct bilirubin, total cholesterol, triglyceride, high density lipoprotein cholesterol(HDL-C), low density lipoprotein cholesterol(LDL-C), urea, serum uric acid (SUA), blood glucose, RBC, WBC, PLTs, Hb, and medical history. These data were used to screen patients who met the inclusion and exclusion criteria in this study.

### PSG Examination

PSG is the gold standard of OSA diagnosis. All participants underwent full overnight PSG (from 21: 00 to 7: 00 the next day) in sleep laboratories of sleep centers of different hospitals (after clinical stabilization during hospitalization). The sleep parameters of all patients were recorded using a laboratory‐based PSG instrument (Compumedics, Melbourne, Australia), including electroencephalography (EEG), electrooculography (EOG), electrocardiography (ECG), nasal-oral airflow, chest and abdominal wall motion, arterial oxygen saturation, and body position [14]. Analysis of sleep tests was carried out according to the American Academy of Sleep Medicine 2017 guidelines [14]. The PSG records of patients in each hospital were manually analyzed and manually calibrated twice by technically certified and experienced technicians, neither of whom had knowledge of demographics and clinical characteristics. Then, a professional sleep physician would analyze and interpret the report. Apnea was defined as the continuous cessation of airflow for more than 10 s, whereas hypopnea was defined as a 30% or greater drop in flow for 10 s or longer associated with ≥ 4% oxygen desaturation (obstructive sleep apnea if respiratory efforts were present) [15]. AHI was defined as the number of apnea and hypopnea events per hour during sleep. OSA severity was defined according to AHI based on the criteria [15] as follows: mild OSA, 5 ≤ AHI < 15; moderate OSA, 15 ≤ AHI < 30; and severe OSA, AHI ≥ 30.

### ECG Examination

For all patients enrolled in the study who were assessed using12-lead ECG or 24-h dynamic ECG, the clinical diagnosis of AF was based on the ACC/AHA/ESC 2016 guidelines [16]. The key electrocardiographic findings of AF are a loss of P waves and replacement by fibrillatory waves; erratic activation of the ventricles resulting in an irregular, rapid heart rate (usually 90 to 170 bpm); and a narrow QRS complex, unless other conduction abnormalities coexist [17].

### Statistical Analysis

All data were analyzed using SPSS version 20.0 (SPSS Inc, Chicago, IL, USA). Metrological data were first tested for normality and homogeneity of variance. Normally distributed metrological data are expressed as the mean ± standard deviation (SD), and one-way analysis of variance or the *t* test was used for comparisons between groups. Metrological data that did not meet the criterion for normal distribution are expressed as the median (interquartile range), and non-parametric tests were used for comparisons between groups. Count data are expressed as percentage (%), and chi-square tests were used for comparisons between groups. Multivariate analyses were performed using binary logistic regression, the results of which are expressed as odds ratio (OR) with 95% confidence interval (CI). The difference was considered statistically significant when *P* < 0.05.

## Results

### Baseline Characteristics

A total of 1285 OSA patients were included for data analysis (Fig. 1), including 305 (23.7%) with mild OSA, 385 (30%) with moderate OSA, and 595 (46.3%) with severe OSA. Age, male, weight, BMI, systolic blood pressure (SBP), AHI, ODI, the time under saturation of 90%, MSpO2, LSpO2, TG, HDL-C, creatinine, SUA, WBC, hypertension, gastroesophageal reflux disease (GERD), coronary heart disease (CHD), carotid atherosclerosis, diabetes mellitus (DM), and paroxysmal AF demonstrated significant differences among groups(*P* < 0.05, Table 1). Compared with non-AF OSA patients, participants diagnosed with AF were found to be older (69.6 VS 66, years), had a higher SBP(140 mmHg VS 131 mmHg), and accounted for a higher proportion of smokers (34.4% VS 21,5%) and drinkers (23.8% VS 11.0%, *P* both < 0.05). The scores for sleep parameters including AHI (32.2 times/h VS 27.2 times/h) and ODI (26.95 times/h VS 22.20 times/h) were higher in AF than non-AF patients (*P* < 0.05), but LSpO2 was lower in the AF group (77% VS 80%, *P* < 0.05). triglyceride (1.32 mmol/L VS 1.39 mmol/L) was lower and creatinine (76 mmol/L VS 72 mmol/L) was higher in AF than non-AF patients(*P* < 0.05). Additionally, those with AF had a higher proportion of CHD (46.7% VS 21.2%), cerebrovascular disease (27.0% VS 16.7%), carotid atherosclerosis (41.8% VS 24.0%), DM (41% VS 23%), peripheral vascular disease (PVD, 9.0% VS 4.5%), chronic obstructive pulmonary disease (COPD,16.4% VS 6.2%), chronic kidney disease (CKD, 9.0% VS 3.5%), and reduced diastolic function (21.3% VS 5.1%, *P* < 0.05); other indexes did not significantly differ between the two groups (Table 2).

**Table 1:** Characteristics of study participants

Indictor	Mild OSA (*n* = 305)	Moderate OSA (*n* = 385)	Severe OSA (*n* = 595)	*P*-value
Demographics				
Age, y	67.00(63.50,72.00)^**b**^	66.00(62.00,72.00)	65.00(62.00,71.00)	0.004
Male,n(%)	168(55.1)^**b**^	231(60.0)^**c**^	395(66.4)	0.003
Height (cm)	165.18 ± 8.02	165.76 ± 7.92	166.48 ± 7.83	0.056
Weight (kg)	69.10 ± 11.77^**b**^	71.30 ± 11.69^**c**^	77.65 ± 12.95	< 0.001
BMI(kg/m2)	25.15(22.66,27.47)^**b**^	25.76(23.63,28.08)^**c**^	27.64(25.25,30.80)	< 0.001
Smoking, n (%)	59(19.3)	81(21.0)	152(25.5)	0.070
Drinking, n (%)	30(9.8)	42(10.9)	85(14.3)	0.100
SBP (mmHg)	130.00(120.00,142.00)^**b**^	130.00(121.50,147.00)	135.00(126.00,147.00)	0.034
DBP (mmHg)	76.00(70.00,80.50)	75.00(70.00,82.00)	76.00(70.00,84.00)	0.156
Sleep parameters				
AHI(events/h)	9.30(6.70,11.90)^**ab**^	21.70(18.10,25.90)^**c**^	47.80(37.30,60.10)	< 0.001
ODI (events/h)	8.30(4.65,11.70)^**ab**^	17.70(12.25,23.35)^**c**^	41.10(29.50,54.20)	< 0.001
TST(h)	7.10(6.27,7.38)	7.11(6.31,7.73)	7.17(6.21,7.79)	0.140
the time under saturation of 90%(min)	3.55(0.70,32.98)^**b**^	10.00(2.25,32.08)^**c**^	32.80(7.70,101.00)	< 0.001
MSpO2(%)	94.00(92.00,95.20)^**b**^	94.00(92.00,95.00)^**c**^	93.00(91.00,94.70)	< 0.001
LSpO2(%)	84.00(80.00,87.00)^**ab**^	81.00(76.00,85.00)^**c**^	75.00(65.00,82.00)	< 0.001
blood index				
TB(μmol/L)	10.60(7.95,14.20)	10.40(8.10,14.35)	10.50(8.10,14.10)	0.963
DB (μmol/L)	3.60(3.00,5.10)	3.50(2.80,4.60)	3.50(2.90,4.80)	0.201
TC(mmol/L)	4.17(3.54,4.99)	4.21(3.57,4.94)	4.28(3.56,4.96)	0.523
TG (mmol/l)	1.36(1.00,1.88)	1.30(0.96,1.81)^**c**^	1.44(1.04,1.96)	0.028
HDL-C (mmol/L)	1.11(0.92,1.40)^**b**^	1.12(0.91,1.40)	1.07(0.88,1.32)	0.023
LDL-C (mmol/L)	2.39(1.89,2.96)	2.41(1.86,2.96)	2.44(1.92,3.03)	0.470
Creatinine(μmoI/L)	68.30(59.5,78.00)^**ab**^	73.00(62.63,85.00)	74.40(63.4,86.30)	< 0.001
Urea (mmol/L)	6.00(4.90,8.70)	6.27(5.00,8.90)	6.30(5.00,8.90)	0.168
SUA(μmol/L)	335.00(282.50,381.00)^**b**^	338.00(290.00,388.07)^**c**^	358.80(313.00,402.00)	< 0.001
Blood glucose(mmol/L)	5.59(5.01,6.43)	5.66(5.06,6.48)	5.71(5.09,6.55)	0.259
RBC(1012/L)	4.49(4.17,4.83)	4.47(4.15,4.78)	4.51(4.22,4.85)	0.280
WBC (109/L)	6.08(5.09,7.13)^**b**^	6.01(5.06,7.06)^**c**^	6.48(5.51,7.42)	< 0.001
PLTs (109/L)	203.00(168.00,239.00)	201.67(169.83,236.00)	205.33(174.92,238.00)	0.174
HB(g/L)	139.20 ± 17.53	138.22 ± 17.72	139.10 ± 18.55	0.887
Medical history, *n* (%)				
Hypertension	190(62.3)^**b**^	231(60.0)^**c**^	411(69.1)	0.009
Hyperlipidemia	96(31.5)	102(26.5)	160(26.9)	0.270
GERD	10(3.3)^**a**^	20(5.2)^**c**^	12(2.0)	0.024
CHD	55(18.0)^**b**^	97(25.2)	152(25.5)	0.030
Cerebrovascular disease	48(15.7)	64(16.6)	115(19.3)	0.333
Carotid atherosclerosis	96(31.5)^**b**^	99(25.7)	135(22.7)	0.017
DM	59(19.3)^**ab**^	93(24.2)	165(27.7)	0.021
Liver disease	11(3.6)	26(6.8)	37(6.2)	0.171
Connective tissue disease	2(0.7)	4(1.0)	6(1.0)	0.834
Peptic ulcer	7(2.3)	8(2.1)	6(1.0)	0.253
PVD	13(4.3)	21(5.5)	29(4.9)	0.771
Solid tumor	15(4.9)	19(4.9)	17(2.9)	0.166
COPD	25(8.2)	31(8.1)	36(6.1)	0.358
CKD	6(2.0)	15(3.9)	31(5.2)	0.064
Reduced diastolic function	19(6.2)	26(6.8)	40(6.7)	0.953
AF				
Paroxysmal AF	10(3.3)*ab*	28(7.3)	55(9.2)	0.008
Persistent AF	11(3.6)	6(1.6)	12(2.0)	

Compare with mild OSA and moderate OSA, ^a^*P* < 0.05; Compare with mild OSA and severe OSA, ^b^*P* < 0.05; Compare with moderate OSA and severe OSA, ^c^*P* < 0.05. *BMI* body mass index, *SBP* systolic blood pressure, *DBP* diastolic blood pressure, *AHI* the apnea–hypopnea index, *ODI* the oxygen desaturation index, *TST* total sleep time, *MSpO2* the mean pulse oxygen saturation, *LSpO2* the lowest pulse oxygen saturation, *TB* total bilirubin, *DB* direct bilirubin, *TC* total cholesterol, *TG* triglyceride, *HDL-C* high density lipoprotein cholesterol, *LDL-C* low density lipoprotein cholesterol, *SUA* Serum uric acid, *RBC* red blood cell, *WBC* white blood cell, *PLTs* platelets, *HB* hemoglobin, *GERD* gastroesophageal reflux disease, *CHD* coronary heart disease, *DM* diabetes mellitus, *PVD* peripheral vascular disease, *COPD* chronic obstructive pulmonary disease, *CKD* chronic kidney disease, *AF* atrial fibrillation

**Table 2:** Characteristics of AF and non-AF participants

Indicator	Non-AF group (*n* = 1163)	AF group (*n* = 122)	*P*-value
Demographics			
Age, y	66.00(62.00, 71.00)	69.60(64.00, 77.00)	** < 0.001**
Male,n(%)	713(61.3)	81(10.2)	0.271
Height (cm)	165.86 ± 7.89	166.89 ± 8.08	0.168
Weight (kg)	73.51 ± 12.74	74.99 ± 13.75	0.229
BMI(kg/m2)	26.49(24.03, 29.05)	26.70(24.22, 30.65)	0.255
Smoking, n (%)	250(21.5)	42(34.4)	**0.001**
Drinking, n (%)	128(11.0)	29(23.8)	** < 0.001**
SBP (mmHg)	131.00(124.00, 144.00)	140.00(125.75, 160.00)	**0.002**
DBP (mmHg)	76.00(70.00,82.00)	77.50(70.00,85.25)	0.163
Sleep parameters			
AHI(events/h)	27.20(15.1, 45.60)	32.20(20.60, 46.83)	**0.044**
ODI (events/h)	22.20(10.70, 40.50)	26.95(17.08, 43.00)	**0.014**
TST(h)	7.11(6.23,7.60)	7.20(6.39,7,71)	0.304
the time under saturation of 90%(min)	15.00(2.56,59.33)	25.15(2.68,73.40)	0.159
MSpO2(%)	93.00(91.80, 95.00)	93.00(91.00, 95.00)	0.112
LSpO2(%)	80.00(72.00, 85.00)	77.00(68.00, 83.00)	**0.005**
blood index			
TB(μmol/L)	10.50(8.10,14.10)	10.85(8.00,14.80)	0.752
DB (μmol/L)	3.60(2.90,4.96)	3.40(2.80,4.40)	0.164
TC(mmol/L)	4.22(3.56,4.96)	4.19(3.44,4.76)	0.233
TG (mmol/l)	1.39(1.02,1.92)	1.32(0.93,1.72)	**0.039**
HDL-C (mmol/L)	1.10(0.89,1.35)	1.11(0.93,1.31)	0.346
LDL-C (mmol/L)	2.42(1.88,3.00)	2.45(1.93,3.05)	0.665
Creatinine(μmoI/L)	72.00(61.75,83.00)	76.00(66.15,88.83)	**0.003**
Urea (mmol/L)	6.22(5.00,8.90)	6.10(4.98,9.03)	0.731
SUA(μmol/L)	343.00(300.00,390.00)	345.55(296.18,408.55)	0.239
Blood glucose(mmol/L)	5.67(5.06,5.67)	5.55(5.03,6.69)	0.833
RBC(1012/L)	4.50 ± 0.56	4.45 ± 0.60	0.393
WBC (109/L)	6.24(5.26,7.19)	6.48(5.48,5.51)	0.141
PLTs (109/L)	204.00(171.00,238.5)	201.00(168.00,235.25)	0.403
HB(g/L)	137.95 ± 17.41	137.07 ± 17.52	0.594
Medical history, n (%)			
Hypertension	750(64.5)	82(67.2)	0.549
Hyperlipidemia	318(27.3)	40(32.8)	0.202
GERD	38(3.3)	4(3.3)	0.995
CHD	247(21.2)	57(46.7)	** < 0.001**
Cerebrovascular disease	194(16.7)	33(27.0)	**0.004**
Carotid atherosclerosis	279(24.0)	51(41.8)	** < 0.001**
DM	267(23.0)	50(41.0)	** < 0.001**
Liver disease	65(5.6)	9(7.4)	0.420
Connective tissue disease	10(0.9)	2(1.6)	0.721
Peptic ulcer	16(1.4)	5(4.1)	0.060
PVD	52(4.5)	11(9.0)	**0.027**
Solid tumor	44(3.8)	7(5.7)	0.419
COPD	72(6.2)	20(16.4)	** < 0.001**
CKD	41(3.5)	11(9.0)	**0.007**
Reduced diastolic function	59(5.1)	26(21.3)	** < 0.001**

*BMI* body mass index, *SBP* systolic blood pressure, *DBP* diastolic blood pressure, *AHI* the apnea–hypopnea index, *ODI* the oxygen desaturation index, *TST* total sleep time, *MSpO2* the mean pulse oxygen saturation, *LSpO2* the lowest pulse oxygen saturation, *TB* total bilirubin, *DB* direct bilirubin, *TC* total cholesterol, *TG* triglyceride, *HDL-C* high density lipoprotein cholesterol, *LDL-C* low density lipoprotein cholesterol, *SUA* Serum uric acid, *RBC* red blood cell, *WBC* white blood cell, *PLTs* platelets, *HB* hemoglobin, *GERD* gastroesophageal reflux disease, *CHD* coronary heart disease, *DM* diabetes mellitus, *PVD* peripheral vascular disease, *COPD* chronic obstructive pulmonary disease, *CKD* chronic kidney disease, *AF* atrial fibrillation

### Prevalence of AF in older patients with OSA

**Fig. 2:**
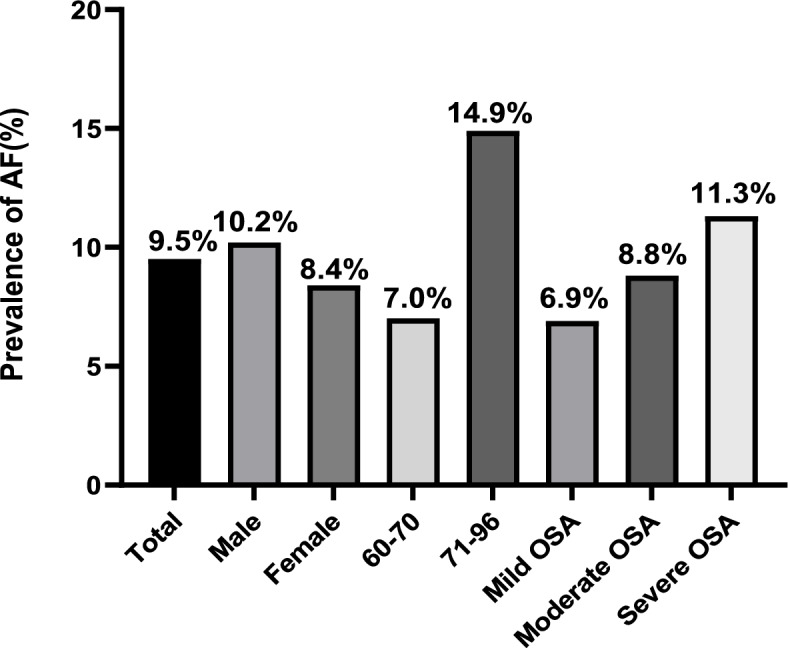
Prevalence of AF in total study population, male and female, different age groups, and severity of AF classes. AF: atrial fibrillation. OSA: obstructive sleep apnea

**Fig. 3:**
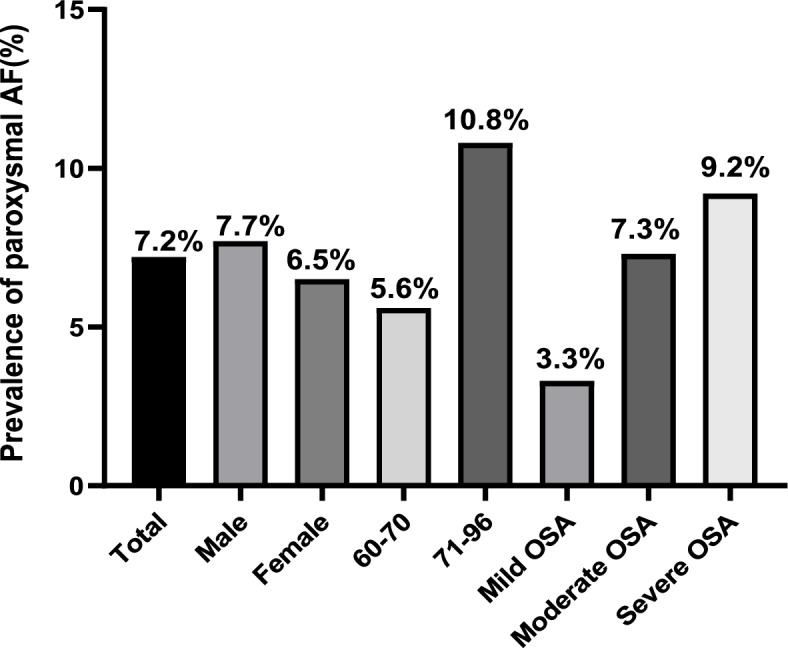
Prevalence of paroxysmal AF in total study population, male and female, different age groups, and severity of AF classes. AF: atrial fibrillation. OSA: obstructive sleep apnea

**Fig. 4:**
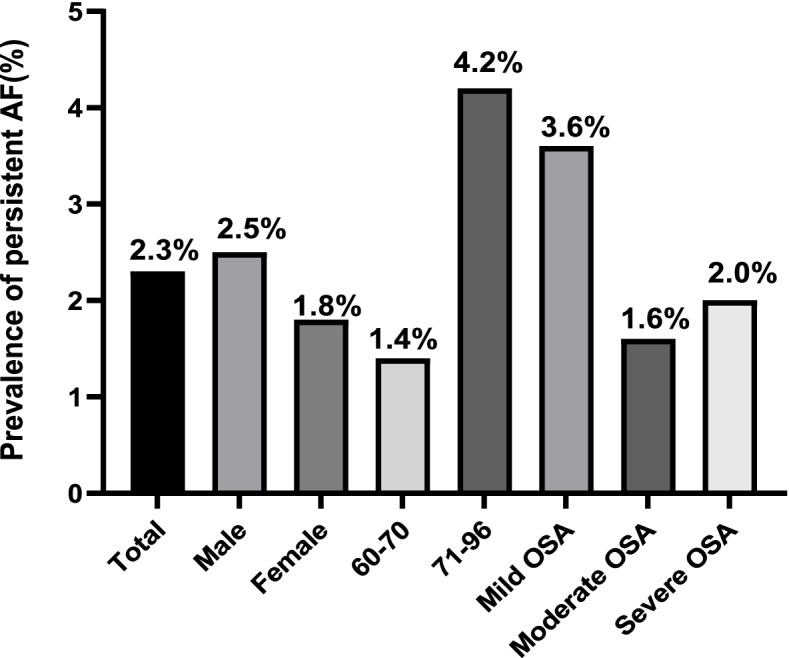
Prevalence of persistent AF in total study population, male and female, different age groups, and severity of AF classes. AF: atrial fibrillation. OSA: obstructive sleep apnea

There were 122 (9.5%) patients with clinically classified AF. Of these, the prevalence of AF in males was higher than that in females: 10.2% in males vs. 8.4% in females. Moreover, the prevalence of AF significantly increased with age: 7.0% in patients aged 60–70 years vs. 14.9% in patients aged 71–96 years. The prevalence of AF tended to increase with the severity of OSA, but there was no significant difference between the three groups **(**Fig. 2**)**. The prevalence of paroxysmal AF was 7.2% among the total study population, and it significantly increased with OSA severity or advanced age (*P* < 0.05). Similarly, the prevalence of AF in males was higher than that in females (7.7% vs. 6.5%; Fig. 3). Furthermore, 2.3% participants were found to have persistent AF, and although the prevalence also increased with age, it did not significantly differ between the mild, moderate, and severe OSA groups (Fig. 4).

### Factors associated with AF in older patients with OSA

In multivariate binary logistic regression analysis, age (OR = 1.055, 95%CI: 1.028–1.082), history of drinking (OR = 1.795, 95%CI: 1.102–2.923), CHD (OR = 1.834, 95%CI: 1.196–2.812), DM (OR = 1.756, 95%CI: 1.163–2.653), and reduced diastolic function (OR = 3.033, 95%CI: 1.757–5.234) were independently related to AF in participants after adjustments for smoking, SBP, AHI, ODI, LSpO2, TG, creatinine, cerebrovascular disease, carotid atherosclerosis, PVD, COPD, and CKD (Table 3).

**Table 3:** Factors associated with AF in older patients with OSA derived from logistic regression with forward stepwise regressive method

	B	S.E	Wals	*P*	OR (95CI)
Age	0.053	0.013	16.117	0.000	1.055(1.028–1.082)
Drinking	0.585	0.249	5.531	0.019	1.795(1.102–2.923)
CHD	0.607	0.218	7.743	0.005	1.834(1.196–2.812)
DM	0.563	0.210	7.166	0.007	1.756(1.163–2.653)
Reduced diastolic function	1.109	0.278	15.870	0.000	3.033(1.757–5.234)

*CHD* coronary heart disease, *DM* diabetes mellitus

## Discussion

Obesity is a well-known major risk factor for OSA [18], although the OSA risk conferred by obesity varies by ethnicity, with the Chinese population being particularly susceptible to weight increases [19]. In 2012, a health survey in Singapore found that, despite having lower body mass index (BMI), the Chinese population had a significantly higher prevalence of OSA (AHI ≥ 15 events/h) than the Indian and Malay population [20]. Furthermore, Chinese participants exhibited greater craniofacial bony restriction than Westerners with a higher BMI [21]. In our study, the median BMI for patients with mild, moderate, and severe OSA was 25.15, 25.76, and 27.64, respectively (3 groups both overweight, *P* < 0.05). A higher BMI was observed in OSA patients with than without AF (median BMI: 26.70 vs. 26.49, *P* > 0.05), but the difference was not statistically significant. In a large observational study of 6841 participants and a follow-up duration of 11.9 years, Cadby et al. [22] found that 455 participants who developed AF were both accepted polysomnography, and the predictors of AF identified on univariate analysis included BMI and, after multivariable adjustment, AHI (HR = 1.55, 95% CI: 1.21–2.00) was identified as an independent predictor of incident AF. There was no correlation between BMI and AF development. Gami et al. [23] found that, in participants younger than 65 years, independent predictors of incident AF included BMI (per 1 kg/m2, HR = 1.07, 95% CI 1.05 -1.10), whereas in participants aged 65 and older, heart failure, but not obesity, was a predictor of incident AF. In our study, the high prevalence of hypertension in OSA patients with and without AF could have resulted in the lack of statistically significant intergroup differences in the prevalence of hypertension. Nonetheless, in our study, the overall systolic BP was higher in OSA patients with AF than in non-AF OSA patients, and this result is in line with the result of the study by Kohno et al. [7].

Among the older participants with OSA in this study, the prevalence of AF was 9.5% and tended to further increase with OSA severity. The abovementioned results differ from those of Hendrikx [24], who reported an AF prevalence of 7.6% in patients suspected to have OSA. Furthermore, in our study, the prevalence of paroxysmal and persistent AF was 7.2% and 2.3%, respectively, and the prevalence of paroxysmal AF increased with the OSA severity. Tanaka et al. [8] reported a 72% prevalence of moderate–severe SA in patients with AF, and that the post-radiofrequency ablation AF recurrence significantly decreased with treatment of OSA [7, 8, 25]. OSA is defined as a partial or complete obstruction of the upper respiratory airway that repetitively causes dyspnea, along with intermittent hypoxemia, hypercapnia, and intrathoracic pressure changes, during nocturnal sleep [8, 26]. Nocturnal hypoxemia in OSA patients prolongs the atrial refractory period and slows atrial conduction, thereby increasing the heterogeneity of the cardiac conduction system. Moreover, repeated nocturnal apnea can promote sympathetic activation and increase the vagal tone to induce or worsen arrhythmia [25, 26]. Stroke, transient ischemic attack, and systemic embolism are the most serious consequences of AF and can significantly increase the mortality and disability rate [27].

Advanced age is a risk factor for both OSA and AF and is associated with dysfunctional and impaired immune regulation [10, 11]. Yao [10] et al. found that the prevalence of AF increases with age, especially in older populations (≥ 60 years). Consistent with the results of the above studies, we found a 5.4% increase in the risk of AF among patients for each additional year of age. Muscle relaxation and muscle strength decline could also be contributing factors in the older people population. A decrease in the upper airway muscle tone can lead to upper airway obstruction, increasing the prevalence of OSA in older people [28, 29]. The routine examination of OSA patients generally excludes 12-lead ECG or 24-h dynamic ECG; therefore, the possibility of AF in OSA patients is overlooked. We should focus on the screening of AF in older patients with OSA to prevent the development of OSA-related cardiovascular and cerebrovascular diseases.

History of drinking significantly contributes to AF occurrence. Drinking is reported to be a definite risk factor for new-onset AF and an independent predictor for recurrence of AF. A meta-analysis elaborated that moderate-severity levels of alcohol consumption are associated with an increase in AF risk, with each 1 SD per day consumed, by approximately 8% [30]. Takigawa [31] et al. demonstrated that the recurrence rate of AF after initial catheter ablation is higher in patients who consumed alcohol than in those who did not (41.9% VS 34.1%). Metabolites of alcohol have direct cardiotoxic effects and indirectly influence cardiac function by leading to disordered breathing during sleep, obesity, or nervous system diseases. Drinking affects the atrial electrophysiological properties, prolonging the intra-atrial and inter-atrial conduction, shortening the right-atrial effective refractory period, and increasing the dispersion of refractoriness, all leading to cardiac electrical remodeling and AF occurrence [32]. Given that the relationship between AF and OSA is well-documented, older OSA patients with binge drinking behaviors are highly prone to AF.

The risk of developing AF is 1.792-fold higher in DM patients than in those without DM, indicating that DM is another factor related to AF development and also closely related to OSA. Staszewsky [33] et al. showed that the risk of AF increases by 32% in patients with DM. Similarly, Papazoglou [34] et al. reported the DM-associated increase in AF risk of 40%. Huang [35]et al. revealed that the prevalence of DM is 16.8% in nonvalvular AF patients. Zhang [36] et al. reported the prevalence of OSA as 60% in hospitalized patients with type 2 DM. DM can affect the prognosis of AF patients, and OSA is closely related to insulin resistance or metabolic disorders. OSA increases sympathetic activity, disrupting the hypothalamus–pituitary–adrenal axis, thereby increasing the risk of metabolic disorders in individuals with OSA. Secondly, intermittent hypoxia in OSA causes oxidative stress, which can directly and indirectly promote inflammation, leading to insulin resistance and impaired beta cell function, setting the stage for DM [37]. Given the close association among OSA, DM, and AF, the prevalence of AF in patients with DM patients was higher than that in patients without DM in this study.

Cardiovascular diseases including CHD and reduced diastolic function were associated with AF among participants. The repeated hypoxemia and hypercapnia in OSA lead to oxidative stress and inflammation. Reactive oxygen species activate nuclear factor-kappa B, which leads to increased production of C-reactive protein, tumor necrosis factor-α, and interleukin-6, along with adhesion molecules such as E selectin, CD15, and CD32 that can cause endothelial damage, CHD, heart failure. And hypoxemia of OSA can decrease the oxygen of cardiomyocytes and cause systolic and diastolic dysfunction [2]. CHD and reduced diastolic function commonly result in abnormal autonomic modulation to affect the stability of cardiac electrophysiology and cardiac pump function to promote the development of AF [38]. It can be seen that cardiovascular diseases played an important role in the AF associated with older OSA patients.

This study was a multicenter, large-sample, explorative observational study, and we have attempted to include as many relevant factors as possible in our analysis. However, there were a few limitations that we assessed the risk factors of in the AF group and the non-AF group of OSA patients without including healthy controls. Secondly, this was a cross-sectional study to investigate the baseline among all participants. consequently, it was difficult to establish a causal relationship between OSA and AF. Finally, the study population was restricted to Chinese, which may have led to unexpected selection bias.

## Conclusion

The prevalence of AF was in older patients with OSA was higher in our study than in similar previous studies. Age, drinking history, CHD, DM, and reduced diastolic function are dependent risk factors for AF. It is important to determine the risk of AF in older patients with OSA because it would reduce the occurrence of major cardiovascular events in these patients.

## Data Availability

The datasets used and/or analysed during the current study available from the corresponding author on reasonable request.

## Abbreviations

OSA: Obstructive sleep apnea
AF: Atrial fibrillation
BMI: Body mass index
SBP: Systolic blood pressure
DBP: Diastolic blood pressure
AHI: The apnea–hypopnea index
ODI: The oxygen desaturation index
TST: Total sleep time
MSpO2: The mean pulse oxygen saturation
LSpO2: The lowest pulse oxygen saturation
TG: Triglyceride
RBC: Red blood cell
WBC: White blood cell
PLTs: Platelets
Hb: Hemoglobin
GERD: Gastroesophageal reflux disease
CHD: Coronary heart disease
DM: Diabetes mellitus
PVD: Peripheral vascular disease
COPD: Chronic obstructive pulmonary disease
CKD: Chronic kidney disease
